# RNA interference of argininosuccinate synthetase restores sensitivity to recombinant arginine deiminase (rADI) in resistant cancer cells

**DOI:** 10.1186/1423-0127-18-25

**Published:** 2011-04-01

**Authors:** Fe-Lin Lin Wu, Yu-Fen Liang, Yuan-Chen Chang, Hao-Hsin Yo, Ming-Feng Wei, Li-Jiuan Shen

**Affiliations:** 1 School of Pharmacy, College of Medicine, National Taiwan University, Taipei, Taiwan; 2 Graduate Institute of Clinical Pharmacy, College of Medicine, National Taiwan University, Taipei, Taiwan; 3 Department of Pharmacy, National Taiwan University Hospital, Taipei, Taiwan; 4 National Center of Excellence for Clinical Trial and Research Center, National Taiwan University Hospital, Taipei, Taiwan

**Keywords:** argininosuccinate synthetase, arginine deiminase, resistance, RNA interference

## Abstract

**Background:**

Sensitivity of cancer cells to recombinant arginine deiminase (rADI) depends on expression of argininosuccinate synthetase (AS), a rate-limiting enzyme in synthesis of arginine from citrulline. To understand the efficiency of RNA interfering of AS in sensitizing the resistant cancer cells to rADI, the down regulation of AS transiently and permanently were performed in vitro, respectively.

**Methods:**

We studied the use of down-regulation of this enzyme by RNA interference in three human cancer cell lines (A375, HeLa, and MCF-7) as a way to restore sensitivity to rADI in resistant cells. The expression of AS at levels of mRNA and protein was determined to understand the effect of RNA interference. Cell viability, cell cycle, and possible mechanism of the restore sensitivity of AS RNA interference in rADI treated cancer cells were evaluated.

**Results:**

AS DNA was present in all cancer cell lines studied, however, the expression of this enzyme at the mRNA and protein level was different. In two rADI-resistant cell lines, one with endogenous AS expression (MCF-7 cells) and one with induced AS expression (HeLa cells), AS small interference RNA (siRNA) inhibited 37-46% of the expression of AS in MCF-7 cells. ASsiRNA did not affect cell viability in MCF-7 which may be due to the certain amount of residual AS protein. In contrast, ASsiRNA down-regulated almost all AS expression in HeLa cells and caused cell death after rADI treatment. Permanently down-regulated AS expression by short hairpin RNA (shRNA) made MCF-7 cells become sensitive to rADI via the inhibition of 4E-BP1-regulated mTOR signaling pathway.

**Conclusions:**

Our results demonstrate that rADI-resistance can be altered via AS RNA interference. Although transient enzyme down-regulation (siRNA) did not affect cell viability in MCF-7 cells, permanent down-regulation (shRNA) overcame the problem of rADI-resistance due to the more efficiency in AS silencing.

## Background

Arginine deiminase depletes arginine by hydrolyzing it to citrulline. Pegylated recombinant arginine deiminase (rADI) has been used as an anti-cancer drug (ADI-SS PEG 20,000 MW) in clinical trials for unresectable hepatocellular carcinoma and metastatic melanoma [1,2]. However, a poor response and resistance to rADI were observed in clinical studies. Only 47% and 25% response rates were observed, respectively, in hepatocellular carcinoma and metastatic melanoma [1,2]. These poor responses indicate that there are obstacles to the clinical application of rADI in cancer therapy.

Argininosuccinate synthetase (AS), a rate-limiting enzyme in the citrulline-arginine regeneration pathway, has been reported to be the crucial enzyme limiting the response to rADI treatment [3,4]. A human melanoma cell line (A375) with no detectable AS expression was sensitive to rADI treatment [4]. In addition, melanoma tissues in patients were found to stain AS-negative prior to rADI treatment; but were found to have become AS-positive as the disease progressed [5]. Our previous study showed that cancer cells with endogenous or induced AS activity (human breast adenocarcinoma MCF-7 and human cervical adenocarcinoma HeLa, respectively) were resistant to rADI [6]. Therefore, if AS confers resistance to rADI, using the RNA silencing technology to down-regulate AS expression might re-sensitize the rADI-resistant cancer cells and overcome the problem of poor response.

RNA silencing, using double-stranded RNA to down-regulate a specific gene, has been used in cancer research *in vitro *and *in vivo *[7]. Short interfering RNA (siRNA) and short hairpin RNA (shRNA) can both be used in RNA silencing technology [8]. However, synthetic 29-mer shRNAs have been reported to have more potency than 21-mer siRNA [9]. In addition, U6 promoter-expressed shRNA, carried by a virus vector, is delivered to the nucleus and amplified by transcription, while siRNA, carried by liposomes, is not amplified intracellularly [10]. Both methods of RNA silencing were used in our study to observe the consequences to cancer cells treated with both rADI and RNA interference to AS expression. Because AS has been reported to play a crucial role in resistance to treatment with rADI in cancer cells *in vitro *and *in vivo*, this study used AS RNA silencing to investigate rADI resistance in cells with endogenous or induced AS expression.

## Methods

### Materials

Recombinant ADI was produced and purified in our laboratory and had an activity of 11.6 U/mg [11]. The micro BCA protein assay reagent kit was purchased from Pierce (Rockford, IL, USA). Lipofectamine™ 2000, Opti-MEM^® ^I Reduced Serum Medium and SuperScript™ II Reverse Transcriptase for RT-PCR were purchased from Invitrogen (Carlsbad, CA, USA). All other chemical reagents were products from Sigma Chemical Company (St. Louis, MO, USA).

### Cell culture

The human breast adenocarcinoma cell line MCF-7, human cervical adenocarcinoma cell line HeLa, and human melanoma cell line A375 were purchased from Bioresource Collection and Research Center (BCRC) in Taiwan (Hsinchu, Taiwan) and maintained in medium recommended by ATCC, supplemented with 10% (v/v) heat-inactivated fetal bovine serum (Invitrogen, Auckland, NZ) and 0.5% penicillin-streptomycin (Invitrogen, Grand Island, NY, USA) in a 5% CO_2_, humidified incubator at 37°C. All other cell culture reagents were products of Invitrogen (Carlsbad, CA, USA).

### Interference of AS expression

#### siRNA

Small interference RNA for the AS gene and the negative control (NC) were designed using a software BLOCK-iT™ RNAi Designer and were synthesized by Invitrogen (Carlsbad, CA, USA). The sequences of the AS gene siRNA (ASsiRNA) and negative control (NCsiRNA) were 5' GCUAUGACGUCAUUGCCUAtt 3' (sense), 5' UAGGCAAUGACGUCAUAGCtt 3' (anti-sense) and 5' GUUUGACUCUCCAAACGGUtt 3' (sense), 5' ACCGUUUGGAGAGUCAAACtt 3' (anti-sense), respectively. MCF-7 and HeLa cells were seeded respectively in culture plates with a density 30% to 50% of confluence and incubated in complete medium without penicillin-streptomycin. For transfection, Lipofectamine™ 2000 was used as suggested by the manufacturer [12]. Western blotting was used to evaluate the effect of ASsiRNA on AS protein in the 1 to 4 days after the transfection of siRNA.

#### shRNA

Lentiviral vectors were produced using pCMV-ΔR8.91, pMD.G, and pLKO.1-shRNA plasmids that carried shRNA against AS mRNA (AS-shRNA: CCGGCCATCCTTTACCATGCTCATTCTCGAGAATGAGCATGGTAAGGATGGATTTTTG) and enhanced green fluorescent protein (EGFP) as control, respectively. All plasmids were co-transfected into 293T cells. Viral particles were harvested from the medium after 40 and 64 hr post-transduction. MCF-7 cells were maintained in RPMI containing 8 μg/mL polybrene and an appropriate amount of virus with multiplicity of infection (MOI) 2.5. After 24 hr viral infection, cells were maintained in RPMI medium with 2 μg/mL puromycin in order to select lentivirus-transduced cells.

### Western blotting

After their respective treatment protocols, cell lysates were prepared according to previous procedures in our laboratory [11]. Samples containing equal amounts of protein were resolved by 10% SDS-polyacrylamide gel electrophoresis (SDS-PAGE) under reduced conditions and transferred to a PVDF membrane (PolyScreen, Boston, MA, USA). The PVDF membrane was blocked with PBST (13.7 mM NaCl, 1 mM Na2HPO4, 0.2 mM KH2PO4, 0.27 mM KCl, 0.2% Tween-20) containing 5% non-fat milk for 1.5 h and then incubated with primary antibodyovernight at 4°C. After the immunoblot was incubated with species-specific horseradish peroxidase (HRP)-labeled secondary antibody for 1 hr at room temperature, the immunoreactive protein bands were visualized using the ECL reagents (PerkinElmer Life Science, Boston, MA) and detected by UVP AutoChemi™ System (UVP, Inc. Upland, CA, USA). The intensity of each band was quantified using UVP LabWork 4.5 software (UVP, Inc. Upland, CA, USA). Signals were normalized according to the expression of the housekeeping enzyme, GAPDH. Antibodies were as follows: AS (Gu-Yuan Biotechnology, Taiwan), PARP-1/2 (H-250)(Santa Cruz Biotechnology, Santa Cruz, CA, USA), α-phospho-AMP kinase (Thr172) (Cell Signaling Technology, Danvers, MA, USA), phospho-4E-BP1 (Thr37/46) (Cell Signaling Technology, Danvers, MA), mouse IgG, and rabbit IgG (Santa Cruz Biotechnology, Santa Cruz, CA, USA).

### PCR for AS DNA and mRNA

#### AS DNA

DNA was extracted from cultured cells using the QIAamp DNA Mini Kit (QIAGEN, Hilden, Germany) and its quality evaluated by agarose gel electrophoresis. PCR primers for AS DNA were 5'ATGGAAGCTGTCTCTGTAGC3' (forward) and 5' CAAGAAGACACACTGGAAGG3' (reverse); and for GAPDH were 5' ACCCACTCCTCCACCTTTGA3' (forward) and 5'CATACCAGGAAATGAGCTTGACAA3' (reverse). The PCR profile condition was: 95°C for 5 min, followed by 35 amplification cycles of 95°C for 40 s, 55°C for 30 s, 72°C for 30 s, and final extension at 72°C for 10 min.

#### AS mRNA

Total RNA was extracted from cells using REzol™ C&T kit (PROtech Technologies Inc., Taipei, Taiwan). First-strand cDNA was synthesized from total RNA using SuperScript™ II RT (Invitrogen). The RT-PCR profile condition was: 42°C for 50 min, and then 70°C for 15 min. Synthesized cDNA was amplified by PCR: the primers of AS were 5'GAGGATGCCTGAATTCTACA3' (forward) and 5'GTTGGTCACCTTCACAGG3' (reverse); and the primers of GAPDH were same as those used for DNA. The PCR profile condition was: 95°C for 5 min, followed by 20 amplification cycles of 95°C for 40 s, 55°C for 30 s, 72°C for 30 s, and final extension at 72°C for 10 min.

### Cell viability assay

Cell cytotoxicity of AS RNA interference and rADI was evaluated by the MTT (3-[4,5-dimethylthiazol-2-yl]-2,5-diphenyl tetrazolium bromide) method [13]. Cells were seeded in 24-well culture plates in MEM medium with supplements and without penicillin-streptomycin. Cells were transfected with ASsiRNA and NCsiRNA with Lipofectamine™ 2000 and treated concurrently with rADI (1 mU/mL). After 1 day-, 2 day-, 3 day-, and 4 day-incubation, 125 μL of MTT stock solution (5 mg/mL) was added to each well and the plates were incubated for an additional 2 hr at 37°. After the discard of the medium containing MTT, the formazan crystal formed in viable cells was solubilized in isopropanol and absorbance at 550 nm was measured.

### Flow cytometry

Analysis of cell-cycle phase distribution in various treatments was evaluated by flow cytometry [14]. After being treated with drugs, cells were harvested with trypsin-EDTA into centrifuge tubes. Cells were centrifuged at 240 g for 10 min to remove supernatant; 70% (V/V) cold alcohol was added to the cell precipitates to fix the cells; and the cells were kept at -20°C. Cells were labeled with propidium iodide (PI) and measured by flow cytometry FACScan FL2 channel and CellQuest program (Becton Dickinson, San Jose, CA, USA).

### Statistical analysis

All values are mean ± SD. Significant difference was evaluated by ANOVA, followed by the Bonferoni modified t-test. Values of p < 0.05 were considered to be statistically significant.

## Results

### Effect of rADI on AS expression in cancer cell lines

**Figure 1:**
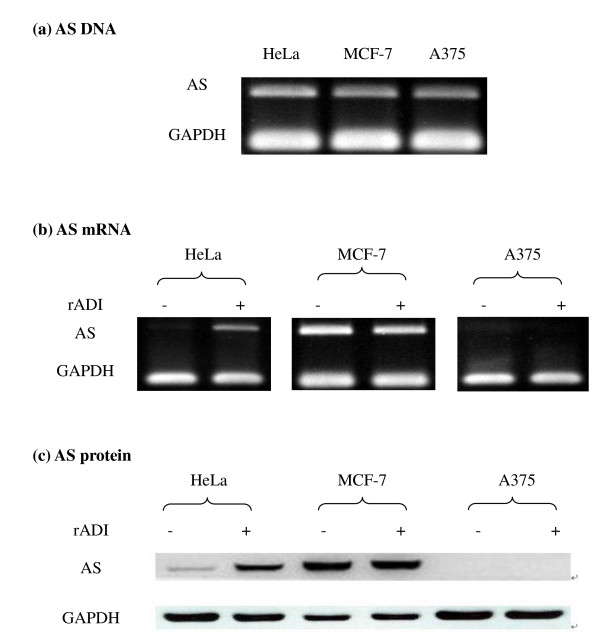
**AS DNA, mRNA, and protein expression in HeLa, MCF-7, and A375 cells**. (a) DAN was extracted from cells, and AS DNA was further amplified by PCR using specific primers. (b and c) Cells were treated with 1 mU/mL of rADI or PBS (as control) for 4 days, and total RNA (b) and protein (c) were extracted. PCR and Western blot were used for evaluation of AS mRNA and protein expression, respectively.

DNA for the AS gene was observed in each of the 3 different human cancer cell lines, HeLa, MCF-7, and A375, used in this study (Figure 1a). Endogenous AS mRNA was detected clearly in MCF-7 cells only when the cells were cultured in the absence of rADI treatment. When cells in the three cell lines were treated with rADI, an increase in AS mRNA (induced AS expression) was seen in HeLa cells, but was not obvious in MCF-7 and A375 cells (Figure 1b). The levels of AS mRNA found in the cells corresponded to the levels of AS protein (Figure 1c). Endogenous AS protein was low in HeLa cells, but induced AS protein was observed clearly in the cells. In MCF-7 cells, endogenous AS protein expression was abundant in the absence of rADI treatment and there was no significant increase in AS expression after these cells were treated with rADI. Expression of AS protein was not detected in A375 cells with or without rADI treatment.

### Down regulation of AS expression by siRNA

#### AS expression

**Figure 2:**
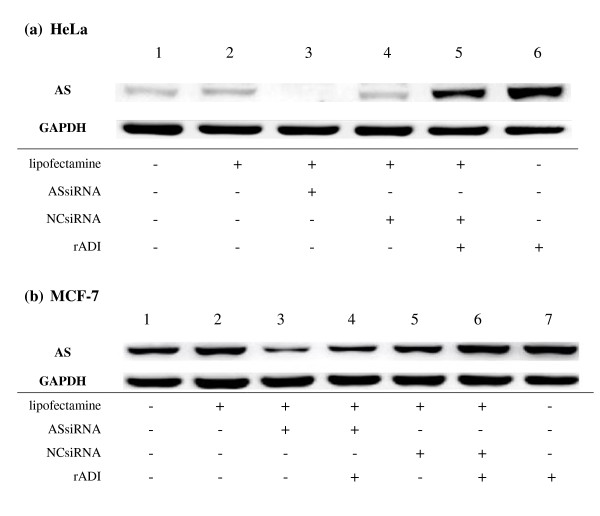
**Effect of ASsiRNA and rADI on AS protein expression in HeLa and MCF-7 Cells**. Cells were seeded in 6-well plates and transfected with ASsiRNA or NCsiRNA by Lipofectamine™ 2000, respectively. After 4-day treatments with different additives, AS protein expression was analyzed both in HeLa (a) and MCF-7 (b) cells. Lipofectamine, ASsiRNA, NCsiRNA, 1 mU/mL of rADI, or combinations of these substances were used. The result of ASsiRNA and rADI in HeLa cells was not present in Figure 2a because of no viable cells after the treatment for western blotting.

When cells were treated with rADI for 4 days, significant amounts of induced and endogenous AS protein were expressed in HeLa and MCF-7 cells (Figures. 2a, Lane 6 and 2b, Lane 7). After ASsiRNA had been transfected into HeLa and MCF-7 cells for 4 days, down-regulation of AS proteins level was seen in both cell types (Figures. 2a, Lane 3 and 2b, Lane 3), but the residual datable amount of AS protein was observed in MCF-7 cells. In contrast, negative control siRNA (NCsiRNA) did not down-regulate AS protein expression in HeLa and MCF-7 cells in the absence or in the presence of rADI (Figure 2a, Lane 4 and 5 and Figure 2b, Lane 5 and 6). AS protein expression in HeLa cells treated with rADI was induced 5.6 ± 2.2 fold (Figure 2a, Lane 1 vs. Figure 2a, Lane 6) that of control without rADI treatment, when normalized by GAPDH expression (p < 0.001). When HeLa cells were treated with ASsiRNA/rADI for 4 days, there were no viable cells in the culture plate for Western blotting. In contrast, when cells were treated NCsiRNA/rADI for 4 days, the cells were viable and the expression of induced AS protein was not significantly different from that seen in rADI treatment alone.

The induction of AS protein expression by rADI treatment, 1.25-fold of control (p > 0.05), was not statistically significant in MCF-7 cells. ASsiRNA significantly inhibited the AS protein expression in MCF-7 cells without and with rADI, to 37% and 46% of each control, respectively. (p < 0.001). There was no effect of lipofectamine and NCsiRNA on AS protein expression in MCF-7 cells in any treatment protocol (p > 0.05).

#### Cell viability and cell cycle

**Figure 3:**
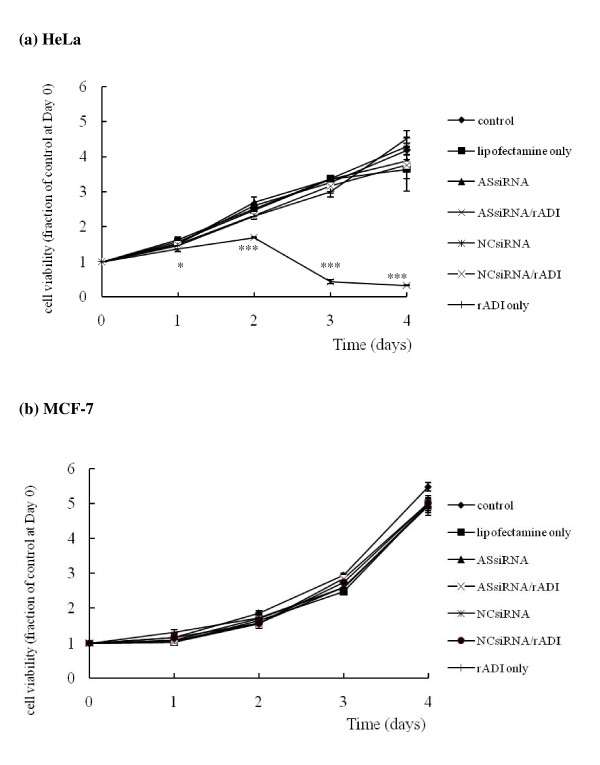
**Effect of ASsiRNA and rADI on viability of HeLa and MCF-7 cells**. Cells were seeded in 24-well plates and treated with different conditions for 4 days, and cell viability was evaluated by MTT assay. These conditions included PBS as control, lipofectamine, ASsiRNA, NCsiRNA, 1 mU/mL of rADI, or combinations of them as shown in the figure. Each group was compared to control on the same day, and the error bars represent standard deviation (n = 6). * p < 0.05, *** p < 0.001.

To observe the effect of the combination of ASsiRNA and rADI in HeLa and MCF-7 cells, cell viability and cell cycle were analyzed by MTT and flow cytometry, respectively. The results of the cell viability (Figure 3) show that only the combination of ASsiRNA and rADI (Figure 3a) significantly inhibited proliferation and survival in HeLa cells. Cell viability was reduced to 90.1 ± 5.1%, 64.9 ± 0.1%, 13.2 ± 1.5%, and 7.7 ± 0.2% of the control after 1, 2, 3, and 4 days of ASsiRNA/rADI treatment in HeLa cells. This phenomenon was only observed in HeLa cells with ASsiRNA/rADI treatment, and not with NCsiRNA/rADI and the other treatments used. In contrast, cell viability in MCF-7 cells was not affected by ASsiRNA/rADI treatment even though AS protein was down-regulated (Figure 3b).

**Figure 4:**
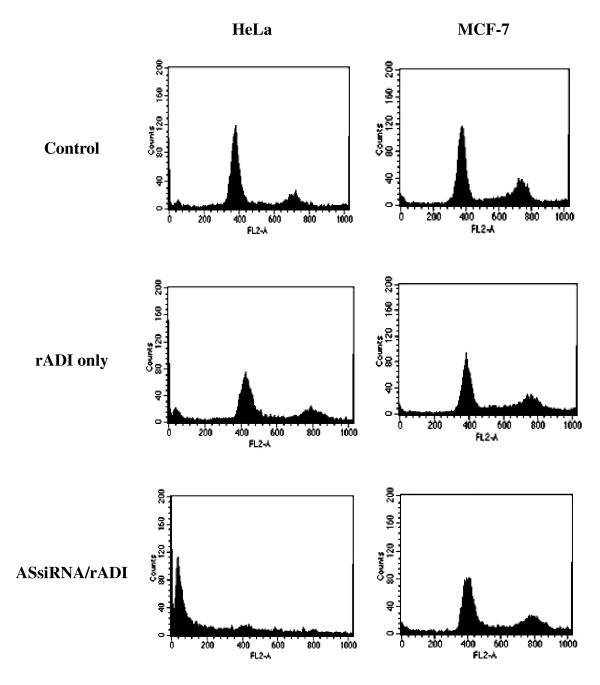
**Effect of ASsiRNA and rADI on cell-cycle phase distribution in HeLa and MCF-7 cells**. Cells were seeded in 6-well plates and collected after treating with PBS as control, 1 mU/mL of rADI, or the combination of ASsiRNA transfection and rADI for 4 days, respectively. The cell collections were stained by propidium iodide and the cell-cycle phase distribution examined by flow cytometry.

The combination of ASsiRNA/rADI influenced the cell cycle in HeLa cells, but not in MCF-7 cells (Figure 4). After 4 days of ASsiRNA transfection and rADI treatment, the percentage of subG1 phase cells increased from 10.7% to 63.4% in HeLa cells.

### Down regulation AS expression by shRNA

ASsiRNA did not effectively down-regulate AS protein expression in MCF-7 cells (Figure 2b). However, shRNA interference with AS protein expression was achieved in MCF-7 cells, using a lentiviral vector to deliver ASshRNA.

#### AS expression

**Figure 5:**
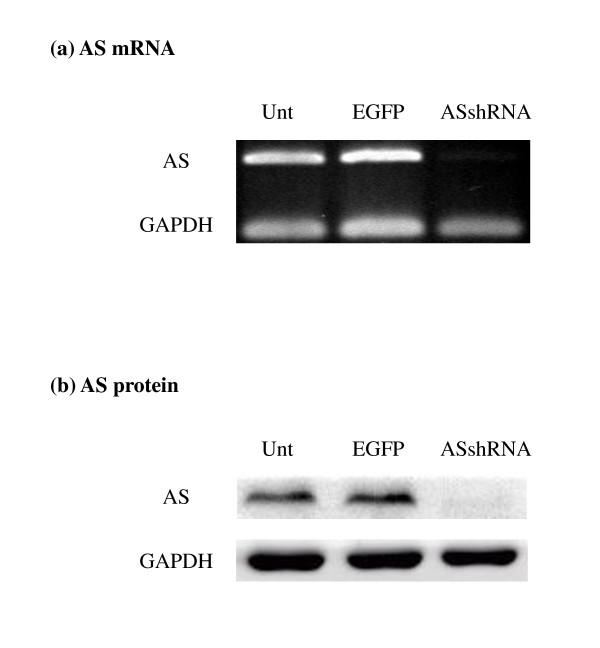
**AS mRNA and protein expression in ASshRNA-tranduced MCF-7 cells**. MCF-7 was transduced with ASshRNA delivered by lentiviruses for generation of stable AS-shRNA expression cell lines. After 15 passages of subculture, total RNA and protein were extracted from the cells. AS mRNA level was evaluated by PCR (a) and endogenous AS protein expression was determined by Western blotting (b). Unt represents untransduced MCF-7; EGFP: EGFP-transduced MCF-7; and ASshRNA: ASshRNA-transduced MCF-7.

Figure 5 shows the results of ASshRNA on AS mRNA and protein expression in MCF-7 cells at the 15th passage after transduction. Compared to the controls (untransduced and EGFP-transduced MCF-7 cells), ASshRNA effectively down-regulated AS mRNA and protein expression due to its specific targeting of AS mRNA. Similar results were observed from the 5th to the 25th passages after puromycin selection.

#### Cell viability and cell cycle

**Figure 6:**
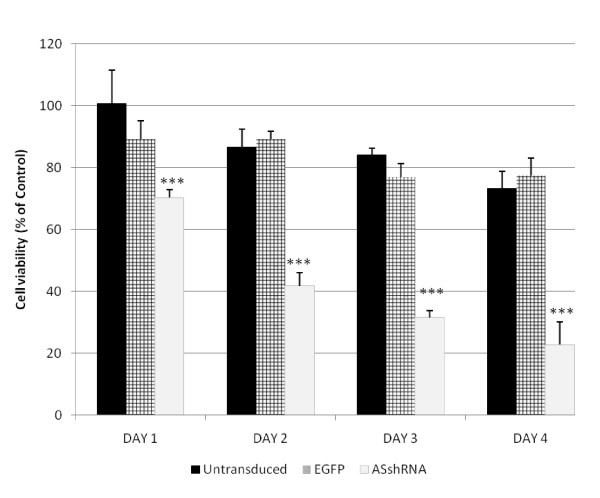
**Effect of ASshRNA interference and rADI on cell viability in MCF-7 cells**. Cells were seeded in 24-well plates and treated with PBS (control) or with 1 mU/mL of rADI for 4 days. The MTT assay was performed to evaluate cell viability. The cells included untransduced MCF-7 (Untransduced), EGFP-transduced MCF-7 (EGFP), and ASshRNA- transduced MCF-7 (ASshRNA). Each group with rADI-treatment was compared to each type of cells in the absence of rADI as control on the same day. Error bars represent standard deviation (n = 6). *** p < 0.001.

Cell viability of untransduced, EGFP-transduced and ASshRNA-transduced MCF-7 cells (control) and with rADI treatment is shown in Figure 6. Cell viability of the untransduced MCF-7 cells after 1 to 4 days treatment with rADI was in the range of 100% to 73% compared to cells without rADI treatment. Similarly, the cell viability of EGFP-transduced MCF-7 cells after 1 to 4 days rADI treatment was 89% to 77% of the controls, a decrease that failed to reach statistical significance. In contrast, the cell viability of ASshRNA-transduced cells under rADI treatment was significantly decreased to 70%, 42%, and 23% of control values on the 1st, 2nd, and 4th days after treatment (p < 0.001).

**Figure 7:**
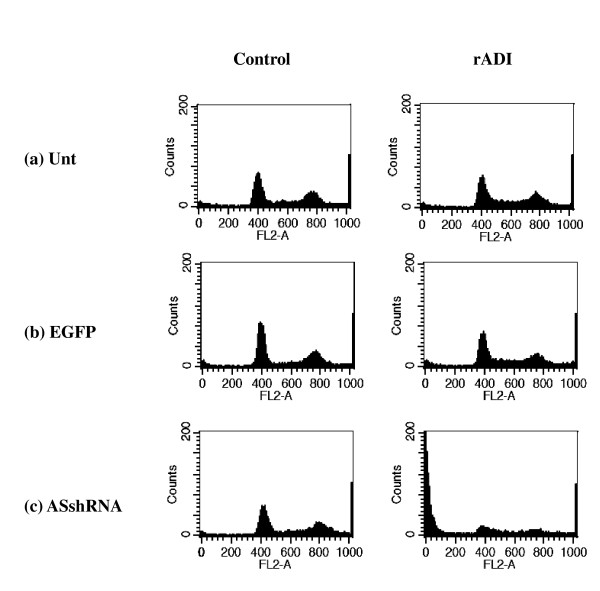
**Effect of ASshRNA interference and rADI on cell-cycle phase distribution in MCF-7**. Cells were seeded in 6-well plates and collected after treatment with PBS (control) or with rADI for 4 days. Collections then were stained with propidium iodide and the cell-cycle phase distribution examined via flow cytometry. Unt represents untransduced MCF-7; EGFP: EGFP-transduced MCF-7; and ASshRNA: ASshRNA transduced-MCF-7.

The effect of rADI on the cell cycle in untransduced, EGFP-transduced, and ASshRNA-transduced MCF-7 cells is shown in Figure 7. The percentages of untransduced MCF-7 cells (the control cells) in the G0/G1 and G2/M phases were 31.9% and 59.3%, respectively, in the absence of rADI treatment (Figure 7a). In addition, fewer than 5% and 10% were in the subG1 and S phases, respectively. A similar cell cycle distribution was seen in untransduced MCF-7 cells in the presence of rADI treatment. The cell cycle patterns of EGFP-transduced MCF-7 cells with and without rADI treatment were also similar to that of the controls (Figure 7b). Although the cell cycle of ASshRNA-transduced MCF-7 cells in the absence of rADI was similar to the controls, significant changes were seen when these cells were treated with rADI. The subG1 phase percentage was increased to 52.7%, and the G0/G1 and G2/M phase percentages were decreased to 12.5% and 30.0%, respectively (Figure 7c).

#### Mechanism of cell death by the rADI and AS protein silencing

**Figure 8:**
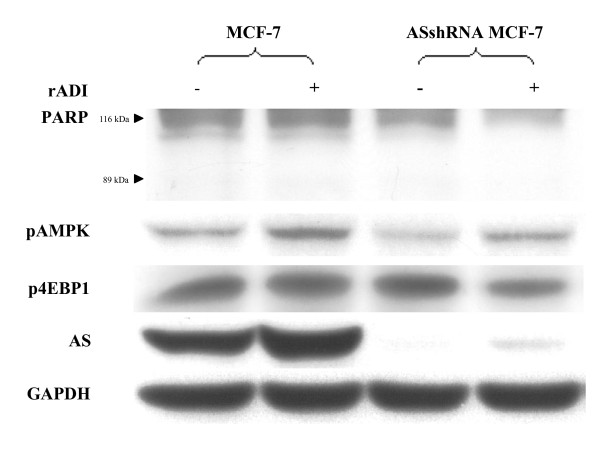
**Effect of rADI on proteins expression involving in pathways of apoptosis**. MCF-7 and ASshRNA-transduced MCF-7 cells were collected after treatment with PBS (control) or rADI for 1 day, respectively. Various proteins, PARP, α-phospho-AMP kinase (pAMPK), phospho-4E-BP1 (p4EBP1), AS, and GAPDH were determined by Western blotting.

To understand the mechanism of rADI causing apoptosis on AS silencing MCF-7 cells, proteins involving in different pathways of apoptosis were analyzed by western blotting in MCF-7 cells and ASshRNA-transduced MCF-7 cells with rADI (Figure 8). After the treatment of rADI, the decreased amount of phospho-4E-BP1 protein expression was observed in ASshRNA-transduced MCF-7 cells, but not in MCF-7 cells. Whereas, rADI caused similar effect on the levels of PARP and phosphor-AMP kinase in MCF-7 cells and ASshRNA-transduced MCF-7 cells (Figure 8).

## Discussion

In this study, the regulation of AS activity by rADI and AS RNA interference was studied in 3 human cancer cell lines. AS DNA was present in all 3 cell lines, but AS expression in mRNA and protein varied. AS expression was undetectable in A375 cells, causing these cells to be sensitive to rADI treatment. According to a previous study [15], the mechanism responsible for the absence of AS expression in cancer cells in spite of the presence of AS DNA might be due to aberrant promoter CpG methylation. The amount of AS protein expression corresponded to the amount of AS mRNA in our results, a finding consistent with other reports [4,15,16]. The AS regulation could be mainly at translational level. In addition, in this report, we also found that induction of AS protein expression by rADI was seen in HeLa cells, causing resistance to rADI treatment in this cell type that has undetectable endogenous AS mRNA.

Cells from two cell lines, HeLa and MCF-7, survived after down-regulation of AS expression only when cells were cultured in complete medium containing arginine. This indicates that AS is not an essential gene in cancer cells when the supply of arginine from extracellular sources is adequate. However, when cells were treated with rADI in the absence of extracellular arginine, the AS gene becomes essential in the AS down-regulated cells. Arginine deprivation in normal cells can block the restriction-point transition, resulting in G1 arrest, a condition in which viability is maintained for extended periods [17], but the same condition in cancer cells leads to cell death on a massive scale within few days [18,19]. Therefore the combination of AS RNA interference and rADI may have selective toxicity toward cancer cells. In our study, we have demonstrated that regulation of AS expression can be a strategy to solve the problem of rADI-resistance in cancer cells. However, further experiments in the targeting of AS RNA interference to tumor cells will be necessary before future clinical application of this strategy is possible.

In our experiments on AS RNA interference, we found ASsiRNA to reduce AS protein expression more efficiently in HeLa cells than in MCF-7 cells (Figure 2). In HeLa cells, but not in MCF-7 cells, AS protein expression was reduced to an undetectable range by ASsiRNA (Figure 3a, Lane 3). We used siRNA mediated by liposomes to knockdown AS gene expression in the rADI-resistant HeLa tumor cell line and then examined the effect of rADI treatment. Introduction of siRNA by this method converted these cells to rADI sensitivity (Figure 3). The HeLa cells thus treated showed DNA damage and a significant increase in the cells in the subG1 phase of cell cycle regulation (Figure 4). This observation shows that the cell death pathway was followed by apoptosis. This result is similar to some reports indicating the ADI that inhibits proliferation of cells by inducing cell cycle arrest and apoptosis [20-22].

Transient AS knockdown with rADI treatment led HeLa cells to die but did not affect the survival of MCF-7 cells even significantly inhibited the AS protein expression to 40% of control. (Figures 3 and 2b). We used ASshRNA carried by lentivirus to transduce MCF-7 cells in order to establish long-term AS gene knockdown and the AS protein expression was in an undetectable level (Figure 5b). When stable AS gene-silenced MCF-7 cells were treated with rADI, cells entered the apoptosis pathway (Figure 7). According to the residual amount of AS protein expression (Figures 2b and 5b), ASshRNA was more efficient than ASsiRNA in the down-regulation of AS expression in MCF-7 cells. Previous reports have shown synthetic 29-mer shRNAs to be more potent inducers of RNA interference than siRNAs [9,23]. When shRNAs delivery is mediated by lentivirus vectors, these RNAs can be delivered into the nucleus and be amplified by RNA polymerase III [24]. In contrast, siRNAs delivered by liposomes are only expressed in the cytosol and therefore cannot be amplified. However, we were unable to explain why the two cell lines, HeLa and MCF-7, respond to siRNA in a different manner. We surmise that differences in the amount of AS protein expressed when protein expression is endogenous protein or induced protein, or some other mechanism, may influence the efficiency of siRNA.

After rADI treatment, the level of phospho-4E-BP1 is decreased in ASshRNA-transduced MCF-7 cells other than in MCF-7 cells (Figure 8). 4E-BP1 plays a crucial role in the mammalian target of rapamycin (mTOR)-mediated translational signaling pathway [25]. A large body of evidence shows that the blockade of mTOR pathways contributes to several anticancer effects, including anti-proliferation and apoptotic cell death [26]. Besides, mTOR pathways are controlled by numerous upstream regulators, such as AMPK and phosphoinositol-3 kinase. The data in the present work support that rADI treatment induces anticancer activity through the inhibition of mTOR-mediated signals but in an AMPK-independent fashion in ASshRNA-transduced MCF-7 cells. However, rADI-treated MCF-7 cells and ASshRNA-transduced MCF-7 cells did not show PARP cleavage, a marker of caspase-dependent apoptosis. It may indicate rADI treatment causes antiproliferation and caspase-independent apoptosis other than caspase-dependent apoptosis. Furthermore, it was reported that the effect of rADI on autophagy was observed in CWR22Rv1 cells expressing undetectable AS protein level, but not in LNCaP cells which express AS protein [22]. We did not observe similar effect of rADI on autophagy in both MCF-cells and ASshRNA-transduced MCF-7 cells by using autophagy inhibitor chloroquine (data not shown). It may be explained by the residual detectable amount of AS expression in ASshRNA-transduced MCF-7 cells. However, autophagy is not normally occurred in a wide variety of cells. Accordingly, the different cell lines may also explain the discrepancy.

## Conclusions

De novo arginine synthesis via the citrulline-arginine regeneration pathway is the determining factor in the success or failure of rADI treatment in cancer [27,28]. Some cancer cells, such as the A375 melanoma cells tested in this study, lack the ability to synthesize arginine de novo via AS and AL [15,29-31] and therefore are sensitive to rADI treatment. However, we found from our results that two prototypes for cancer cells, HeLa and MCF-7, were resistant to rADI treatment. Cell types similar to HeLa cells have low endogenous AS protein expression but conspicuously induced AS protein expression after rADI treatment. Cell types like MCF-7 cells have abundant endogenous AS protein expression and do not show visibly induced AS protein expression after rADI treatment. We have also demonstrated that AS down-regulation can change rADI-resistant into rADI-sensitive cancer cells. The mechanism of rADI on anticancer effect in ASshRNA-transduced MCF-7 cells may involve the inhibition of 4E-BP1-regulated mTOR signaling pathways. Different efficiency in AS down-regulation by siRNA or shRNA was observed in HeLa and MCF-7 cells. These findings will be important to treatment outcome when rADI is introduced into cancer therapy.

## Competing interests

The authors declare that they have no competing interests.

## Authors' contributions

FLW and LJS conceived of the study, and participate in its design and coordination and helped to draft the manuscript. YFL and YCC carried out the AS shRNA and siRNA studies, respectively. HHY prepared and recombinant protein arginine deiminase for studies. MFW studied the mechanism of apoptosis by the treatment of ASshRNA/rADI in MCF-7 cells. All authors read and approved the final manuscript.
